# Projected changes in climate extremes over Tanzania

**DOI:** 10.1038/s41598-024-79432-w

**Published:** 2025-01-02

**Authors:** Philbert Modest Luhunga

**Affiliations:** Department of Documentation and Publication, Directorate of Knowledge Management, Commission for Science and Technology, Dar Es Salaam, Tanzania

**Keywords:** Climate extreme, Climate indices, Regional climate models, CORDEX, Tanzania, Climate sciences, Climate-change impacts

## Abstract

Understanding projected changes in climate extremes at local and regional scales is critical for reducing society’s vulnerability to such extremes, as it helps to devise informed adaptation strategies and contributes to informed decision-making processes. In this paper, we analysed projected changes in climate extremes across regions in Tanzania using outputs of high-resolution regional climate models from the Coordinated Regional Climate Downscaling Experiment program (CORDEX-Africa). The indices analysed here are those recommended by the Expert Team on Climate Change Detection and Indices (ETCCDI) to characterise climate extremes over different regions. The results revealed that Tanzania would experience an increased number of warm days and nights during the present (2011–2040), mid (2041–2070), and end centuries under the RCP4.5 emission scenarios. Further, projections reveal that in future climate conditions, heavy, very heavy and exceptionally heavy rainfall events would dominate over regions along coast, central regions, northwestern parts and southwestern and northeastern highland.The number of consecutive wet days (CWDs) are likely to increase across large areas of Tanzania and more rapidily over coastal regions than that in other regions for all seasons. However, many regions in Tanzania are likely to experience an unchanged to decreasing number of consecutive dry days (CDDs). Areas along coastal regions would experience increased intensity and frequency of extreme rainfall events in the present, mid, and end centuries under the RCP4.5 emission scenario. These increases in extreme climate events are likely to pose significant damage to property, destruction of infrastructure, and other socioeconomic livelihoods for people in many regions of Tanzania. It is therefore recommended that appropriate policies are put in place to help different sectors and communities at large adapt the impacts of climate change in the future climate under RCP 4.5 scenario.

## Introduction

The components of the Earth’s climate system, including the atmosphere, lithosphere, biosphere, hydrosphere, and cryosphere, are undergoing rapid and unprecedented changes. As these components are interconnected, any change in one component could cause negative/positive feedbacks, long-lived change, or devastating impacts on other climate components.

The reports of three working groups of the recent sixth Intergovernmental Panel on Climate Change (IPCC) Assessment Report (AR6) indicated that humans play a central role in modifying the global climate^1^. In particular, a report from working group one (WGI) on the physical science basis of climate change showed unequivocally that human activities have altered the composition of the atmosphere by pumping more greenhouse gases such as carbon dioxide (CO2), nitrous oxide (N2O), methane (CH4) and sulfur oxide (SOx)^2^. These human-induced greenhouse gases have amplified the natural greenhouse effect and thus increased the near-surface temperature to 1 °C, above the 1850–1900 reference period^3^. The increases in global near-surface temperature as a result of increased human-induced greenhouse gases in the atmosphere have drastically affected the frequency, intensity, duration, and severity of climate extremes such as tropical cyclones, heatwaves, droughts, and floods in every region and every continent and cause huge socioeconomic losses across the world^4^. These climate extreme events are already causing significant loss of life and impacting biodiversity, infrastructure, human health, and agriculture. They have exposed millions of people to acute food and water insecurity, particularly in Africa, Asia, Central and South America, small islands, and the Arctic^3,5,6^.

Taking a global perspective, the World Meteorological Organization’s State of the Global Climate 2023 highlights how increased global temperatures contribute to more frequent, intense, and longer-lasting marine heat waves in the worldwide ocean^4^. These heatwaves have far-reaching consequences for aquatic life and the communities that depend on them. They also influence intense cyclones, leading to severe weather and socioeconomic losses. Indeed, according to^7^, the tendency of increased global temperatures to trigger El Niño events is becoming stronger and more frequent. As a result, regions like Southeast Asia and southern Mexico, northern South America, and South Africa experienced drier than usual conditions and wetter than normal conditions in parts of Chile and East Africa.

The rise in global temperatures has impacted the drivers of weather and climate extreme events across timescales ranging from days to several months in some cases and regions leading to occurance of compound extreme^8^. Notably, phenomena such as the Indian Ocean Dipole (IOD), El Niño–Southern Oscillation (ENSO), and the North Atlantic Oscillation (NAO) are currently undergoing unprecedented changes to influence short-term climate variability worldwide^9,10^. Tanzania is experiencing unprecedented changes in the patterns, duration, and intensity of the factors that influence the local climate. For instance, it has been reported that the intertropical convergence zone (ITCZ), which plays a significant role in controlling the climate of Tanzania, has become shallow in recent years, and the vertical mean ascent of wind that influences deep convection activities along the ITCZ has weakened. It has also been reported that there is a rapid passage of the ITCZ across Tanzania, affecting the convergence of monsoon wind systems. This has affected the start and cessation of the rainy season. It has also resulted in climate extremes such as floods, droughts, and increased dry and wet spell alterations that occur simultaneously at intraseasonal, interseasonal, and interannual scales, causing cascading impacts on multiple sectors, such as agriculture, livestock, transportation, energy, environment, infrastructure, health, and water resources.

Studies show that climate extremes will increase in the future, which is consistent with global warming^11,12^. The projected global warming of 1.5 °C in the next two decades (year 2040) will result in more climate extremes that will present serious challenges to contemporary adaptation strategies, and in some regions of the world, it will be impossible to adapt to the impacts of climate extremes if global warming exceeds 2 °C^2^. In this regard, the international community has invested in implementing the Paris climate change agreement, which aims to maintain the increase in global warming relative to preindustrial levels, well below 2 °C, and proposed a major ambition target of 1.5 °C to save the global climate^13^.

The near-surface temperature of Tanzania is projected to increase by 2 °C in 2040, by 3 °C in 2070, and by 5 °C in 2100^14^. This increase in surface temperatures relative to the (1971–2000) reference period is likely to further increase the frequency and intensity of climate extremes. An increase in climate extremes will result in additional severe socioeconomic damage, some of which could be irreversible for adaptation. This will present significant challenges to efforts to achieve sustainable development goals (SDGs)^15^.

One of the strategies to reduce vulnerability to extreme climate events is to characterize the climate extremes in the future climate to help the government and the general public in planning adaptation strategy decisions. The Intergovernmental Panel on Climate Change (IPCC) has a role in providing reports about the past and future global climate. However, their assessment analysis is comprehensive to assess climate change at global and continental levels. There is limited country-specific information in the IPCC reports that provide detailed analysis of historical and future projections of climate extremes that are adequate for impact assessment at the country and sectoral levels^16,17^. This study aims to provide valuable high-resolution information about future (2011–2100) climate extremes across regions of Tanzania using high-resolution climate simulations derived from the Coordinated Regional Climate Downscaling Experiment Program (CORDEX). Thus, information from this study about future climate extremes in Tanzania has the potential to be used to update the policy and decision framework about adaptation to climate extremes across various sectors. The main sections of this manuscript are structured as follows: Sect. 1 presents the introduction about climate extremes (linking climate extremes to increased temperature). Section 2 presents the data and methodology used in this study, Sect. 3 presents the main results, Sect. 4 presents a discussion, and Sect. 5 presents conclusions and recommendations.

## Data and methodology

### Study region

**Fig. 1:**
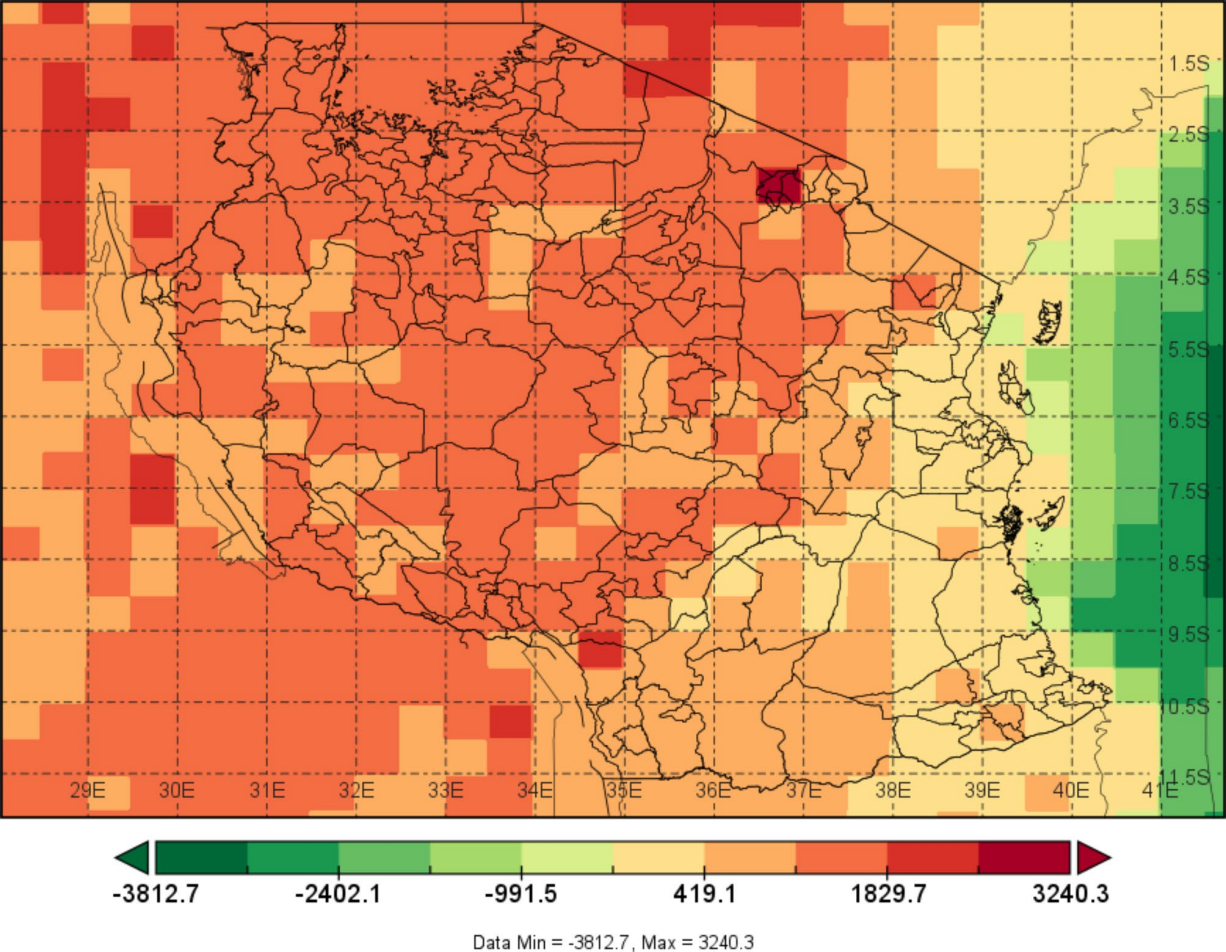
The topographical map (elevation in m) showing international and administrative district boundaries of Tanzania. The Shapefile for elevation was obtained from global digital elevation model (DEM) GTOPO30 available at https://dds.cr.usgs.gov/ee-data/coveragemaps/shp/ee/gtopo30/gtopo30.zip and the Shapefile for administrative boundaries were obtained from Tanzania National Bureau of Statistics at https://www.nbs.go.tz/statistics/topic/gis.

The study area is Tanzania, which is located in East Africa between longitudes 29°E to 41°E and latitudes 1°S and 12°S (Fig. 1). The country shares borders with Uganda and Kenya in the North, Burundi, Rwanda, and the Democratic Republic of Congo in the West, Malawi and Zambia in the Southwest, Mozambique in the South and the Indian Ocean in the East.

The country has complex topographic terrain that plays a significant role in influencing heterogeneity in the region’s climate. Regions in the northern and northern coasts, the island of Zanzibar (Pemba and Unguja) and the northeastern highlands receive bimodal rainfall patterns, while regions in the central and southern coast, southern, western, and southwestern highlands receive unimodal rainfall patterns^18^. These rainfall patterns are mainly driven by the movement of the Inter Tropical Convergence Zone (ITCZ)^19^. This zone moves from north to south in Tanzania from October to February and from south to north in Tanzania from March to May.

The average seasonal rainfall in Tanzania ranges from 50 to 200 mm per month, with high variations between regions; during the wettest seasons, some regions may receive as much as 300 mm of rainfall per month. The annual average temperature and rainfall across regions of Tanzania range from 14.4 to 26.4 °C and 534 to 1837 mm, respectively^19^. Compared with other regions, the western and coastal regions experience higher temperatures. The season with high temperatures across the regions starts in October and continues through February or March. The season with low temperatures across the regions starts in May and continues through August or September. The annual average minimum and maximum temperatures across the regions ranged from 9.6 to 22 °C and from 19.1 to 30.7 °C, respectively.

### Data

#### Model and observed Data

Model data: This study uses climate simulations from the Coordinated Regional Climate Downscaling Experiment (CORDEX) program. This is an international collaborative project that was pioneered by the World Climate Research Program (WRCP) to produce dynamically downscaled climate simulations for model intercomparison and climate change impact assessment around the world. CORDEX-RCM outputs are archived and distributed by the Earth System Grid Federation (ESG) using software that allows users to access all the archived data in a more friendly and transparent manner^20^. This study uses climate simulations accessed from https://esg-dn1.nsc.liu.se/projects/esgf-liu/website. These datasets have been quality controlled and may be used according to the terms of the use document found at https://cordex.org/data-access. Table 1 summarises the information about the CORDEX regional climate models and their driving GCMs used in this study.

**Table 1:** The CORDEX-Regional Climate models (RCMs) and their driving general circulation models (GCMs).

No.	RCM-name	Model Centre	Short name of RCM	Resolution	GCM	GCM model centre
1	High-Resolution limited Area Model version 5	Danmarks Meteorologiske Institut(DMI), Danmark	HIRHAM5	Rotated pole 0.44°	EC-EARTH	EC-EARTH consortium
2	Rossby Center Regional Atmospheric Model version 4	Sveriges Meteorologiska och Hydrologiska Institut (SMHI), Sweden	RCA4	Rotated pole 0.44°	EC-EARTH	EC-EARTH consortium
					MPI Earth System Model running on a low-resolution grid	Max Planck Institute for Meteorology (MPI-M)
					Centre National Recherches Météorologiques Coupled Model 5 (CNRM)	Centre National de Recherches Météorologiques/Coupled Model 5 Centre Européen de Recherche et Formation (CERFACS)Avancée en Calcul Scientifique
3	Regional Atmospheric Climate Model, version 2.2	Koninklijk Nederlands Meteorologisch Instituut (KNMI), Netherlands	RACMO22T	Rotated pole 0.44°	EC-EARTH	EC-EARTH consortium

Daily rainfall, minimum, and maximum temperature datasets from three high-resolution CORDEX regional climate models (RCMs) driven by three general circulation models (GCMs) for the reference period (1971–2000) and future climate projections (2011–2100) under representative concentration pathway (RCP4.5) are used in the computation of climate extremes.

Observed data: In addition to climate simulation datasets from CORDEX-RCMs, this study uses gridded climate reanalysis data from the Physical Sciences Laboratory (PSL). These datasets are created by merging climate observations from many different sources, such as ships, satellites, ground stations, radiosondes, and radar. The outputs from merged climate data are then gridded at different spatial and temporal resolutions using the Shepard algorithm^21^. Daily rainfall and minimum and maximum temperatures for a thirty-year climatological period (1971–2000) interpolated at 0.5° over a global spatial grid are used to statistically downscale CORDEX regional climate model outputs to high spatial resolutions.

### Methodology

Studies^19,22^, have indicated that CORDEX RCMs systematically fail to reproduce the climatological patterns of rainfall in different areas of Tanzania. In areas that receive a bimodal pattern of rainfall, the CORDEX RCMs systematically underestimate rainfall amounts in the March-April-May (MAM) season and systematically overestimate the amount of rainfall in the October-November-December (OND) season. However, in areas with unimodal rainfall patterns, the CORDEX RCMs fail to reproduce the length of the rainy season and overestimate the rainfall from September to January. On the other hand, the CORDEX RCMs underestimate the magnitude of the maximum temperature and fairly capture the magnitude of the minimum temperature.

The failure of the CORDEX RCMs to correctly simulate the seasonal cycles of climate variables in Tanzania, where the models fail to capture the phase and magnitude of seasonal rainfall, and temperature might introduce uncertainties in the calculated climate extremes. Moreover, all CORDEX RCMs are set to simulate climate variables at a spatial resolution of 0.44° by 0.44°, which is approximately 50 km by 50 km. This spatial resolution is still too small to provide useful information about climate extremes for impact studies on biodiversity, ecosystem services, agricultural systems, species distribution, conservation planning, and other landscape and agriculture-related matters^23,24^. In this study, we employ two approaches to address the uncertainties that could be introduced in the computation of climate extremes by the inability of the CORDEX RCM to reproduce the seasonal cycles of climate variables, but at the same time, we downscale the CORDEX RCM to a higher spatial resolution. The first step is to construct an ensemble average of five RCMs-GCMs combinations for thirty (30) years (1971–2000) of historical climatological data and for future (2011–2100) climate projections under representative concentration pathway (RCP) scenarios: RCP4.5. Thus, taking into account the five RCM-GCM combinations under RCP 4.5 emission scenario, we have five ensemble members for analysing climate extremes in the future climate of Tanzania. The second step is to transfer the CORDEX RCM model simulations from 0.44° by 0.44°, which is approximately 50 km by 50 km, to 0.05° to 0.05°, which is approximately 6 km by 6 km in spatial resolution using the inverse distance weighting interpolation technique^19^.

The climate simulatiton from CORDEX RCM were Statistically downscaled using delta method^25^. The relevance and weakness of the method include the stationarity problem which assume the predictand and predictor relationship remain the same in historical climate as in future climate under emission scenarios. However^25^, evaluated types of statistical downscaling techniques and projection of climate extremes in central Texas, USA and found that delta downscaling technique adjust climate simulated model data for computation of climate extremes.

The delta for downscaling temperature is calculated using the following steps, (i) using model simulated data to compute change in temperature for the present (2011–2040), mid-2041-2070), and end (2071–2100) centuries under the RCP4.5 emission scenario relative to the historical (1971–2000) climate simulation. (ii) The change in temperatures in present, mid and end centuries climatological periods were added to the historical climate observations (1971–2000) to obtain the corrected climate simulation in the respective climatological period. This method can be represented mathematically as follows:

1 $$\:{\nabla\:{T}_{d\:Future\:i}=T}_{d\:Future\:i}-{T}_{d\:Baseline\:or\:reference}$$

2 $$\:{TC}_{d\:Future\:i}={T}_{d\:Observed\:in\:reference\:climate}+\nabla\:{T}_{d\:Future\:i}$$

where $\:\nabla\:{T}_{d\:Future\:i}$ is the change in the simulated daily temperature in the future $\:i$ climatological period, such as the present, middle and end centuries, relative to the historical climate simulation.

$\:{T}_{d\:Future\:i}$ is the simulated daily temperature in the future $\:i$ climatological period

$\:{T}_{d\:Baseline\:or\:reference}$ is the simulated daily temperature in the historical period

$\:{TC}_{d\:Future\:i}$ is the corrected daily temperature for the future $\:i$climatological period

The delta for downscaling rainfall simulation was calculated as a ratio of observed rainfall data and simulated rainfall data derived from the ensemble average of five RCMs in the historical (1971–2000) climate. The calculated delta is used to downscale the simulated rainfall in the specified future climate projection. Mathematically, the method is represented as follows:

3 $$\:{RDelta}_{d,baseline}=\frac{{R}_{d,O}}{{Rd,M}_{}}$$

4 $$\:{RC}_{d,i}={R}_{d,i}\times\:{RDelta}_{d,baseline}$$

where $\:{R}_{d,O}$ is the daily rainfall observed during the historical period, $\:{R}_{d,M}$ is the daily rainfall from the model simulation during the historical climate period, $\:{RDelta}_{d,baseline}$ is the ratio of observed and simulated daily rainfall during the historical period, and $\:{RC}_{d,i}$ is the corrected rainfall simulation for the future $\:i$climatological period.

The downscaled climate variables (rainfall and minimum and maximum temperatures) are used to compute extreme climate indices. The extreme climate indices computed in this study are recommended by the Expert Team on Climate Change Detection and Indices (ETCCDM) for characterising climate extremes across regions. The first group of indices computed in this study includes percentile-based indices such as the percentage of cold nights (TN10p), percentage of warm nights (TN90p), percentage of cold days (TX10p), percentage of warm days (TX90p), percentage of very wet days (which represent the amount of rainfall falling above the 95th percentile (R95p)) and percentage of extreme wet days (which represents the amount of rainfall falling above the 99th percentile (R99p)).

The second group of indices computed are the absolute indices that represent the minimum or maximum values of climate variables within a season or year. These include the maximum daily maximum temperature (TXx), maximum daily minimum temperature (TNx), minimum daily maximum temperature (TXn), minimum daily minimum temperature (TNn), maximum 1-day precipitation amount (RX1day) and maximum 5-day precipitation amount (RX5day).

The third category includes threshold-based climate indices. These indices are the number of days on which a climate variable such as temperature or precipitation falls above or below the fixed threshold. These indices include the number of heavy precipitation days > 10 mm (R10) and the number of very heavy precipitation days > 20 mm (R20). The fourth category of indices is duration-based indices, which define periods of excessive warmth, cold, and wetness. These indices include the length of the longest dry spell in a year (CDD) and the longest wet spell in a year (CWD).

The future changes in extreme climate event indices were analysed by calculating the differences between the extreme climate event indices in the present (2011–2040), middle (2041–2070) and late (2071–2100) centuries under two emission scenarios (RCP4.5) relative to the baseline period (1971–2000).

## Results

This section presents the results that are analysed and presented in two subsections. The first subsection presents the analysis of downscaled CORDEX climate simulations across regions of Tanzania. The climate extremes relate to temperatures deduced from percentile based indices such as the number of cold days (TX10p) and cold nights (TN10p), and the number of warm days (TX90p) and warm nights (TN90p), the climate extremes relates to rainfall deduced from percentile based indices such as very wet days (95th percentile) and extreme wet days (99th percentile). The projections of heavy, very heavy and exceptionally heavy rainfall categories together with threshold based indices such as consecutive dy days (CDD) and the consecutive wet days (CWD) are presented in subsection two.

### Downscaled CORDEX climate simulation across the region of Tanzania

**Fig. 2:**
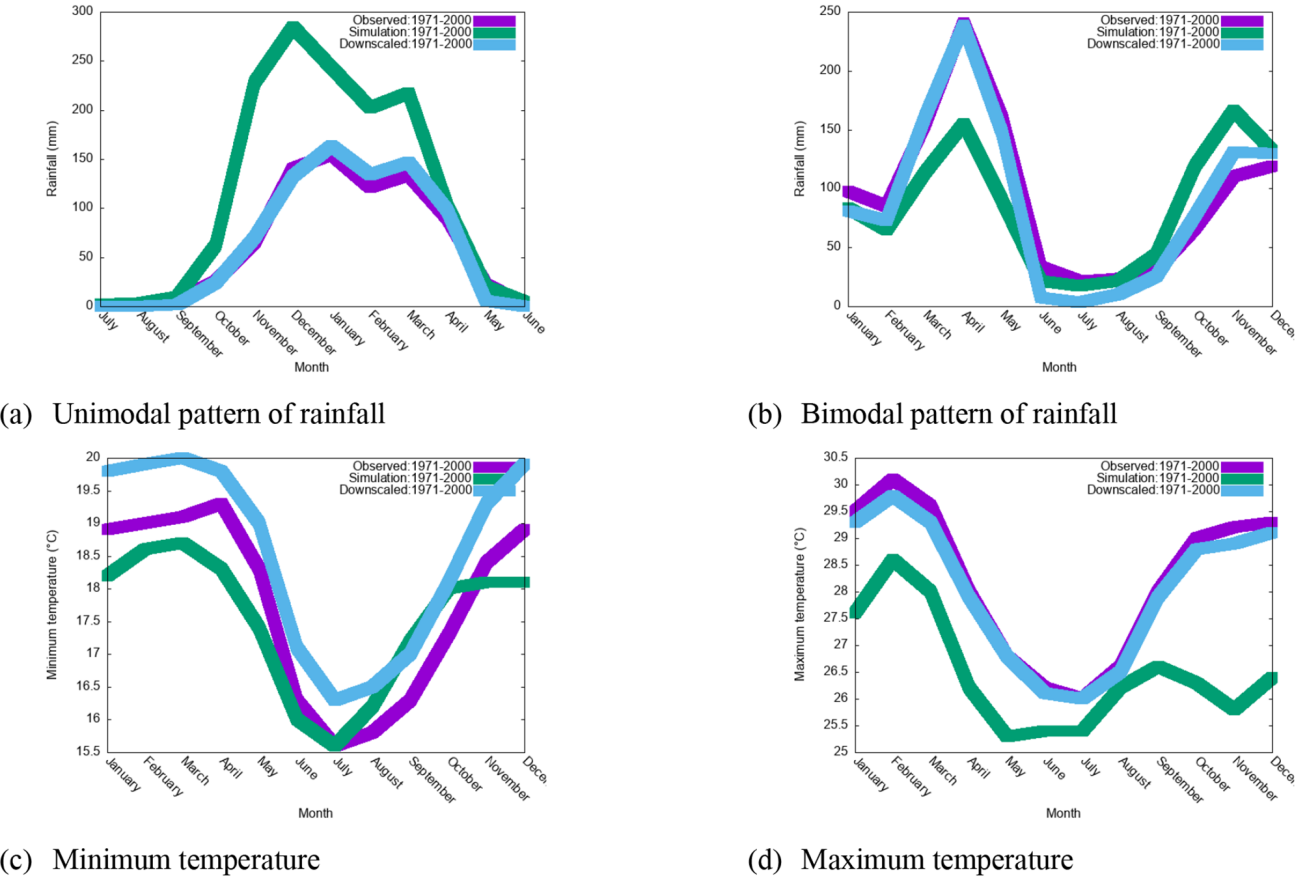
Observed and simulated annual cycles of rainfall and temperature calculated as time averages across all grid points in Tanzania from 1971–2000.

Figure 2 presents the annual cycle of rainfall and temperatures in Tanzania under the historical (1971–2000) climate. Figure 2 clearly shows that CORDEX RCMs systematically fail to reproduce historical climate patterns of rainfall over areas receiving both Bimobal and unimodal patterns of rainfall. For instance, over areas receiving a bimodal pattern of rainfall, the ensemble of three CORDEX RCMs driven by three GCMs systematically underestimates the amount of rainfall in the March-April-May (MAM) season and systematically overestimates the amount of rainfall in the October-November-December season. However, downscaled climate simulations from an ensemble of RCMs captured well climatological rainfall over regions receiving both bimodal and unimodal patterns of rainfall. Therefore, the overall finding here is that climate simulations derived directly from RCMs often do not represent the observed regional climate, and statistical downscaling must be applied to adjust the data to obtain realistic regional rainfall and temperature patterns. This finding is in agreement with that of^26^, who argued that the results from RCMs often fail to represent local or regional climates and that statistical empirical schemes must be applied to refine the data to obtain a realistic regional or local representation of the climate.

### Climate extremes that relate to temperature

#### Analysis of the number of warm days (TX90p) and cold days (TX10p)

**Fig. 3:**
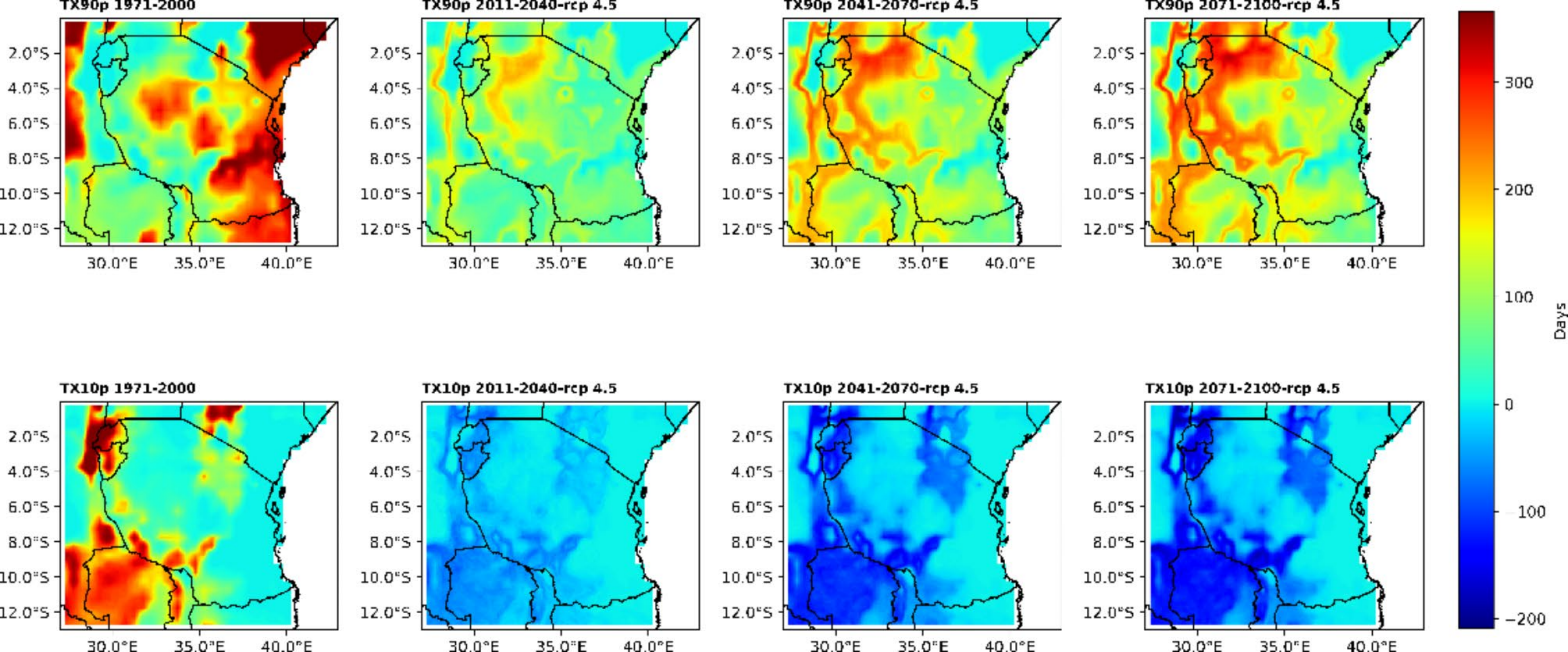
The upper panel shows the spatial distribution of the number of warm days, defined as a temperature greater than or equal to 30 °C, and the bottom panel shows the number of cold days, defined as a temperature less than or equal to 20 °C.

The spatial patterns of the number of warm days (TX90p) and cold days (TX10p) under historical and future climate conditions are presented in Fig. 3. It can be seen from the figure that climatologically (1971–2000), warm days (TX90p) are greater over the entire coast, extending inland to central parts of Tanzania. The number of cold days (TX10p) is greater in the western region, eastern part of Lake Nyasa, northeastern region, and southwestern highlands.

The historical climatological patterns described above, however, are likely to change in the future climate. For instance, under the RCP4.5 scenario, the hottest climatic zones are projected to expand across regions in Tanzania. This expansion is likely to be greater in the western and northern regions than in the coastal regions. In nearly all the western regions, the northern and southwestern highlands are likely to warm faster than other regions in the future climate (Fig. 3). The analysis further reveals that the western and northern regions are likely to warm more rapidly in the mid-century under RCP4.5 than in the present century. However, in the end century, more regions across Tanzania, particularly in the western and northern parts, are likely to warm more rapidly than in the middle and present centuries.

On the other hand, in the future climate under the RCP4.5 scenario, the number of cold days is projected to decrease across regions of Tanzania. This decline is likely to be greater in colder regions than in warmer regions. Overall, these findings imply that under the RCP4.5 emission scenario, colder regions such as the southwestern and northeastern highlands are likely to wam more rapidly than warmer regions such as coastal regions (Fig. 3). Notably, in most regions, the number of cold days is projected to decrease more rapidly from the present to the end of the century.

#### Analysis of the number of warm nights (TN90p) and cold nights (TN10p)

**Fig. 4:**
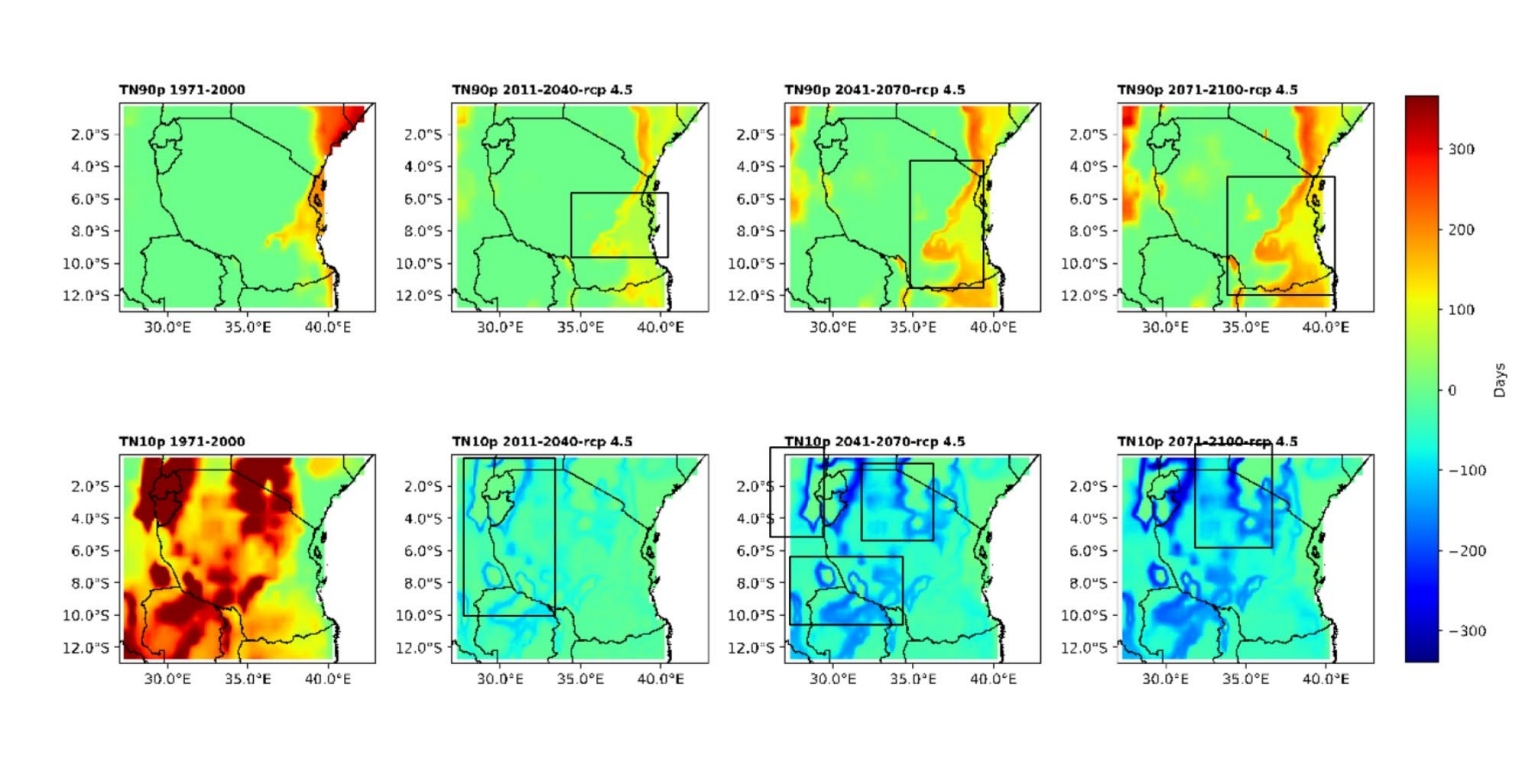
The upper panel shows the spatial distribution of warm nights defined by a temperature greater than or equal to 25 °C, and the bottom panel shows the number of cold nights defined by a temperature less than 15 °C.

Figure 4 presents the spatial patterns of the number of warm nights (TN90p) and cold nights (TN10) under historical (1971–2000) and future climate conditions. It is clear from the figure that climatologically (1971–2000), warm nights (TN90p) are greater over coastal regions extending to regions that are nearby and along the coast. However, under the RCP4.5 scenario, the number of hot nights will continue to increase across all regions but will increase more rapidly along the coast. Overall, under the RCP4.5 emission scenario, in the future climate, areas with warmer nights, such as regions along the coast, are likely to wam more rapidly than other regions. It is important to also note that in coastal regions, the number of warm nights is projected to increase more rapidly from the present to the end of the century.

The historical number of cold nights (TN10p) (1971–2000) revealed that the northwestern, northeastern and southwestern highlands had more cold nights when compared to other regions of Tanzania. However, under RCP4.5, cold nights are likely to decline more rapidly in the western region and in the northeastern and southwestern highlands. Overall, areas that historically had colder nights are decreasing rapidly. This decrease is projected to increase more rapidly from the present to the end of the century. These results support what has been concluded by the fourth IPCC assessment report that anthropogenic greenhouse gas influences changes in global temperature (very likely)^27^. This will influence countries to experience a decreased number of both cold days and nights (very likely)^27^.

### Climate extremes that relate to raifall

#### Analysis of duration-based indices the consecutive dry days (CDD) and consecutive wet days (CWD)

**Fig. 5:**
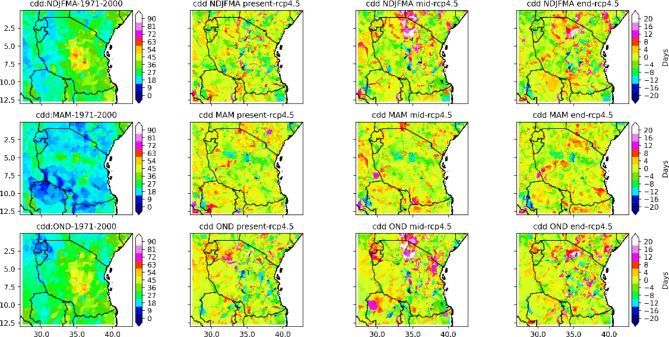
Spatial distributions of consecutive_dry_days_index_per_time_periods in the present, middle and end centuries under the RCP4.5 emission scenario compared to the historical (1971–2000) climate.

**Fig. 6:**
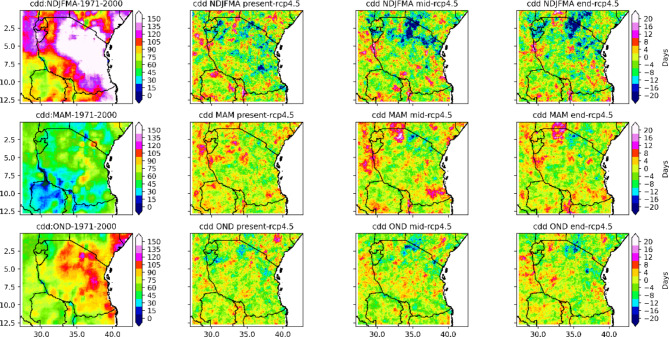
Spatial distributions of number_of_cdd_periods_with_more_than_5days_per_time_period in the end, middle and present centuries under the RCP4.5 emissions scenario relative to the historical period (1971–2000).

The seasonal means of the consecutive dry days index per time period (CDDs) as well as the CDDs periods with more than 5 days per time period are depicted in Figs. 5 and 6, respectively. Projections indicate that CDD with less than 1 mm is expected to increase in isolated areas and regions across Tanzania. For instance, in the present century, under the RCP4.5 scenario, based on the results presented in Fig. 5, in the NDJFMA season, CDD is projected to increase by approximately 3 to 12 days, predominantly in the northern coast, western regions, southern part of Lake Victoria, and southwestern highlands, while isolated places over the northeastern highlands and eastern part of Lake Victoria are likely to experience increased CDD of approximately 12 to 27 days (see Fig. 5). On the other hand, as shown in Fig. 5, in the present century, under RCP 4.5, in the NDJFMA season, many areas across Tanzania, particularly in the southern part of the central region, western region, northeastern highlands and southern region, are likely to experience no change in CDD, decreasing from approximately − 3 days to -18 days. The projected CDD periods of more than 5 days per period increase by approximately 3 to 27 days in a few isolated areas in the eastern part of Lake Victoria, northeastern parts of the country and southwestern highlands. However, under RCP4.5, projected CDD periods of more than 5 days per time period in the NDJFMA season for the present century are projected to remain unchanged and decrease for approximately 0 to -24 days in many regions of the central-southern parts, northeastern highlands, northern coast and western and southern parts of Lake Victoria. The overall findings imply that in the NDJFMA season, for the present century, under the RCP4.5 scenario, many regions of Tanzania are likely to experience an unchanged to decrease in both the consecutive dry days index per time period (CDD) and the CDD periods of more than 5 days per time period (see Figs. 5 and 6).

In the NDJFMA season, for the mid- and end-century (2041–2070) under the RCP4.5 scenario, large areas of the southeastern part of Lake Victoria, the northern coast of a particular coastal region and the Dar es Salaam, central-northeastern highlands, are likely to experience an increase in CDD of approximately 5 to 40 days. On the other hand, based on the presented results in Fig. 5, the projected CDD periods of more than 5 days per period increase by approximately 3 to 27 days in a few isolated areas in the southwestern highlands, northwestern parts and parts of the southern coast during the NDJFMA season for the middle and end centuries under the RCP4.5 scenario. As shown in Fig. 5, in the middle and end centuries, under RCP 4.5, in the NDJFMA season, many areas across Tanzania, in particular, the western, southern, and northern coasts, southern coast and parts of the northeastern and southwestern highlands, are likely to feature CDD decreases of approximately zero to -30 days. The projected CDD periods of more than 5 days per time period in the NDJFMA season for the mid-century under RCP 4.5 are shown to remain unchanged and decrease for approximately 0 to -35 days in many regions of the northern, northeastern highland and northern coast; central-southern parts and southern coast; and part of the western region. These results suggest that in the NDJFMA season, for the middle and end centuries, under the RCP4.5 scenario, many regions of Tanzania are likely to experience an unchanged to decrease in both the CDD index per time period and the number of CDD periods of more than 5 days per time period.

The results of the projected consecutive dry days index per time period (CDD) as well as the CDD periods with more than 5 days per time period in the MAM season for the present century under the RCP4.5 scenario reveal that both the CDD and CDD periods with more than 5 days per period are expected to increase in a few areas and regions across Tanzania. These regions include parts of southern and coastal regions, limited areas over western regions, southern parts of Lake Victoria, central regions and southwestern highlands that are likely to experience increased CDD and CDD periods of more than 5 days per period of approximately 2 to 12 days and 2 to 18 days, respectively (see Fig. 5). However, as shown in Fig. 5, in the present century, under RCP4.5, in the MAM season, many areas across Tanzania, in particular, the central regions, northeastern highlands, western regions, southern regions, and southern coast, are likely to experience unchanged to decreased CDD of about Zero to -22 days. In addition, the projected CDD periods of more than 5 days per period in the MAM season for the present century under RCP4.5 are shown to remain unchanged and decrease for approximately 0 to -15 days in many regions of the central-southern parts, northeastern highlands, northern coast and western and southern parts of Lake Victoria. The overall findings imply that in the MAM season, for the present century, under the RCP4.5 scenario, many regions of Tanzania are likely to experience an unchanged decrease in both the consecutive dry days index per time period (CDD) and the CDD periods of more than 5 days per time period (see Figs. 5 and 6). In the mid- and end-century, under RCP 4.5, a few areas on the northern and southern coast, the western parts of Lake Victoria and the western and southern regions will experience increased CDD and CDD periods of more than 5 days per time period, both of about 3 to 27 days. However, many regions across Tanzania are projected to feature unchanged to decreased CDD of approximately zero to -20 days.

In the OND season, for the present century, under RCP4.5, the regions that receive a bimodal pattern of rainfall, parts of the northeastern highlands and parts of the northern coast, northern region, southern and western parts of Lake Victoria, and northwestern regions are likely to feature CDD in the range of 5 to 20 days. The southwestern highlands and western parts of Lake Nyasa are likely to experience an increase in CDD per time period and CDD periods of more than 5 days per time period of approximately 5 to 25 days and 2 to 15 days, respectively. In the OND season, in the mid- and end-century, under the RCP4.5 scenario, the projections shown in Figs. 5 and 6 indicate that a large area of Tanzania will experience CDD periods, with more than 5 days per time period ranging from zero to 40 days and from zero to 20 days, respectively.

**Fig. 7:**
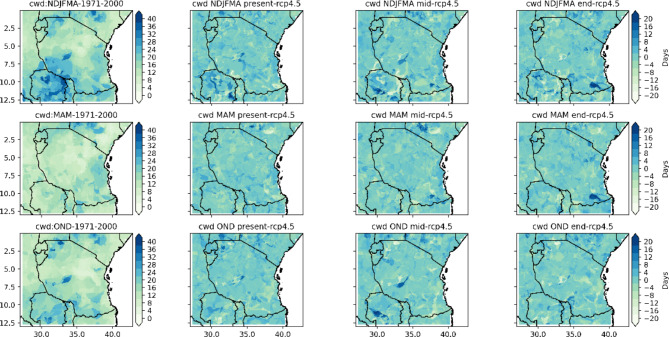
Spatial distribution of consecutive_wet_days_index_per_time_period in the end, middle and present centuries under the RCP4.5 emissions scenario relative to the historical period (1971–2000).

**Fig. 8:**
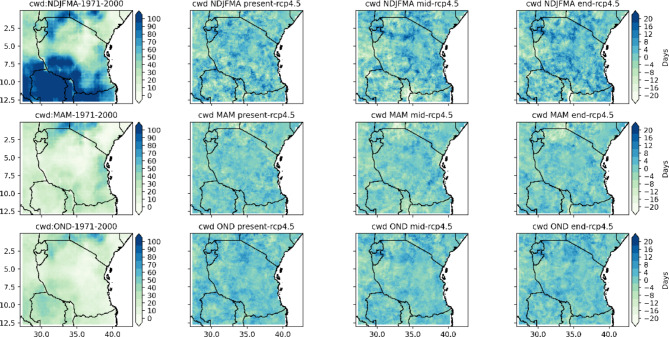
Spatial distribution of number_of_cwd_periods_with_more_than_5days_per_time_period in the end, middle and present centuries under the RCP4.5 emissions scenario relative to the historical period (1971–2000).

**Fig. 9:**
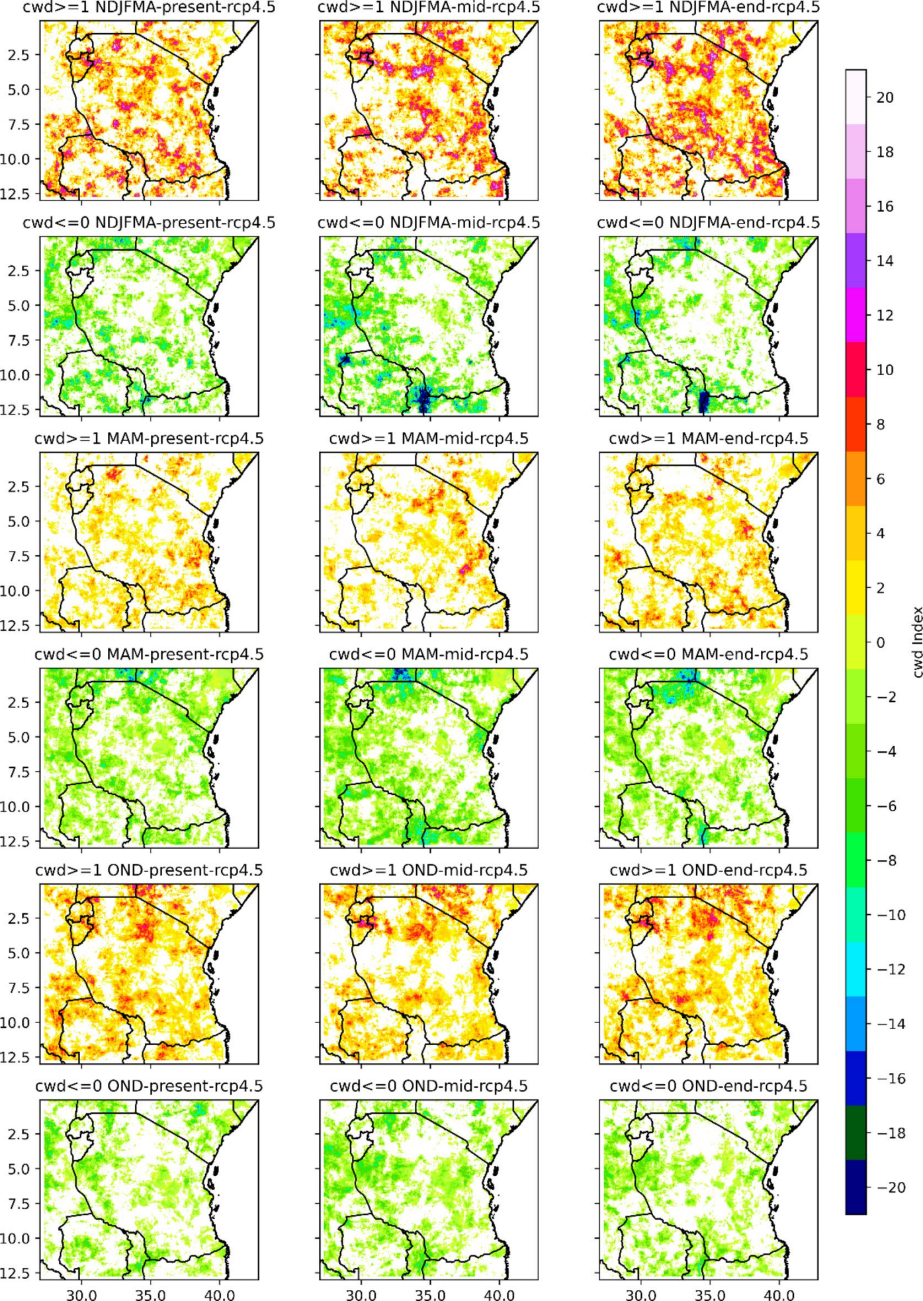
Spatial variation of projected changes in seasonal consecutive wet days across regions in Tanzania.

Figures 7 and 8 show the results of the projected consecutive wet days index (CWD) per time period and the number of CWD periods of more than 5 days per time period with reference to the base (1971–2000) period for Tanzania based on the RCP4.5 scenario. The CWD is defined as the largest number of consecutive wet days of a time series of daily rainfall amounts greater than 1 mm. The seasonal means of the CWD per time period and the CWD periods of more than 5 days per time period shown in Figs. 7 and 8 reveal that the coastal southwestern and northeastern highlands, western and southern regions, southern parts of Lake Victoria, and the northeastern highlands to central regions of Tanzania are projected to have more CWD per time period under the RCP4.5 scenario. The number of CWD periods of more than 5 days per time period is projected to increase by approximately 2 to 20 days in the coastal regions, central and western parts of Lake Victoria and southern and northeastern highlands for the present century under RCP 4.5. However, for regions across Tanzania in the southwestern highlands and southern, central, and southeastern regions of Lake Victoria, the northwestern parts are likely to experience unchanged to decreases in the CWD per time period of approximately zero to -24 days under RCP 4.5 for the present century. However, isolated areas of Tanzania are likely to feature unchanged to decreasing numbers of CWD periods of more than 5 days per time period of approximately zero to -13 days for the NDJFMA season under the RCP4.5 scenario. In all coastal regions, the CWD index per time period is projected to increase more rapidly than that in other regions in the middle and end of the century for all seasons under RCP4.5. Additionally, as shown in Fig. 8, the number of CWD periods with more than 5 days per time period is projected to increase by 2 to 22 days for the entire coastal region and the central and northeastern highlands. Figure 9 indicates CWD in different seasons are likely to increase across regions in Tanzania.

#### Analysis of percentile-based indices represented by the 95th and 99th percentiles of daily rainfall

**Fig. 10:**
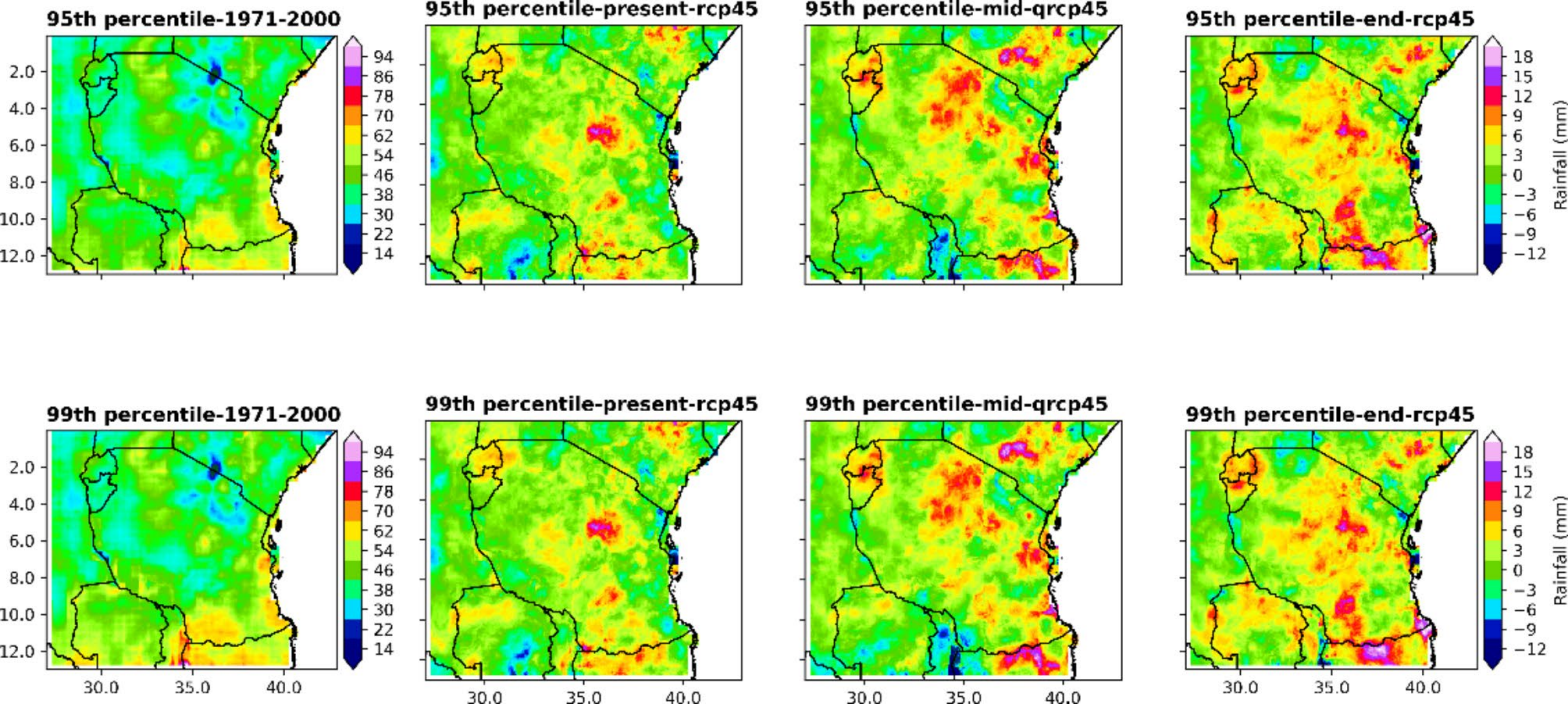
Spatial variation in the 95th and 99th percentiles of rainfall indicating heavy rainfall and extreme rainfall amounts, respectively, in the end, middle and present centuries under the RCP4.5 emission scenario relative to the historical scenario (1971–2000).

Projections of the 95th and 99th rainfall percentiles of Tanzania computed using daily rainfall with reference to the base (1971–2000) period under the RCP4.5 scenario are depicted in Fig. 10. The 95th and 99th percentiles are higher in the central, southern and western regions in the present century under the RCP4.5 scenario. In the mid- and end-century, under the RCP4.5 scenario, both the 95th and 99th rainfall percentiles are likely to increase by approximately 3th to 12th percentiles, especially in the northeastern highlands, central regions, coastal regions, southwestern parts of Lake Victoria, and southwestern highlands. These findings imply that in the central parts, the northeastern highlands and coastal regions and southwestern highlands are likely to experience heavy to extreme rainfall for all periods under the RCP4.5 scenario.

**Fig. 11:**
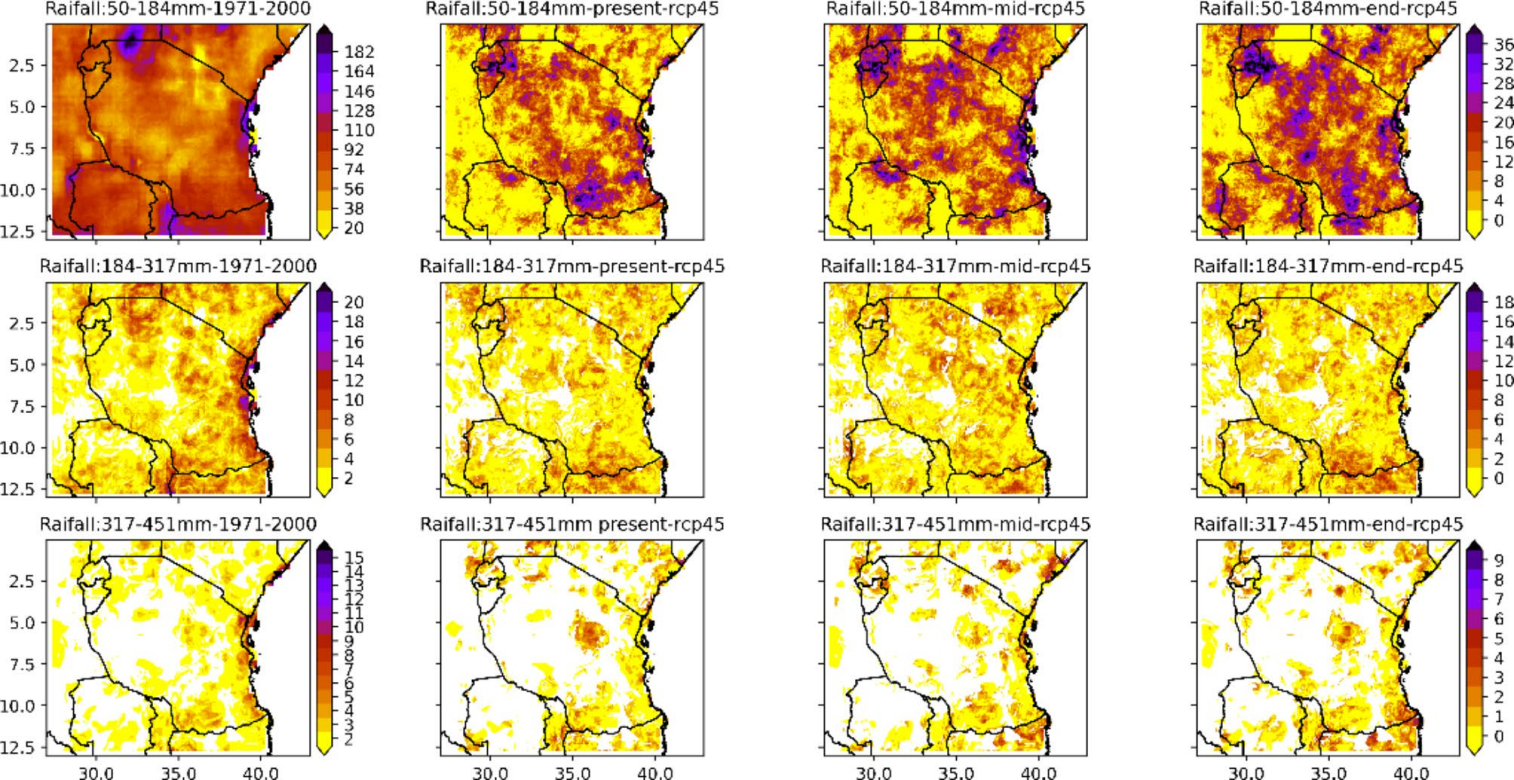
The spatial distribution of heavy to exceptionally heavy rainfall in Tanzania in the present, middle and end centuries relative to the refence time (1971–2000).

The projected changes in heavy (50–184 mm), very heavy (184–317 mm) and exceptionally heavy rainfall (317–451 mm) for three time periods regarding the base perido under RCP4.5 are depicted in Fig. 11. In this figure, the left panel column corresponds to heavy, very heavy and exceptionally heavy rainfall across regions in Tanzania, and the second and last columns correspond to projected changes in heavy, very heavy and exceptionally heavy rainfall under 4.5. It is important to note that the categories of heavy, very heavy and exceptionally heavy rainfall distributions presented in this paper were chosen subjectively based on comparisons of the rainfall category intervals and the associated socioeconomic impacts of communities in Tanzania. The historical impacts of heavy rainfall events and associated impacts are published annually by the Tanzania Meteorological Authority (TMA) in the Annual Statement of Tanzania Climate. Here, we calculated and categorised extreme rainfall into three groups using the distribution of the observed extreme rainfall across Tanzania, as reported in the statements of Tanzania climate since 2011 to 2023, where the maximum amount of rainfall in Tanzania across meteorological stations was 450.7 recorded in Pemba in 1978 and the lowest extreme that caused destruction of properties and death was 50 mm. The class width was computed using the formulation suggested by^28^ as *n* = 1 + 3.3*log10(N), where n is the number of data points used for estimation.

Figure 11 shows that the entire country is projected to experience increased extreme rainfall events for all projected time periods under the 4.5 emissions scenario. The projections indicate that in the present, mid- and end-century, under the RCP4.5 scenario, the northeastern parts of the country, southwestern highlands, coastal regions and northeastern highlands are likely to experience an increase in heavy rainfall of approximately 12 to 36 days for all projected periods. The amount of very heavy rainfall is likely to increase across regions in Tanzania, particularly over coastal regions and southern and northern regions where an increase in the amount of heavy rainfall of approximately 2 to 10 days is likely to dominate in all projected time periods. It is projected that all coastal regions, parts of the southwestern and northeastern highlands, are likely to experience an increased amount of exceptionally heavy rainfall in all projected time periods under RCP4.5. These results imply that in future climate conditions, under RCP4.5, heavy, very heavy and exceptionally heavy rainfall events are likely to dominate regions along coastal regions (e.g., the northern and southern coasts), central regions, northwestern parts, and southwestern and northeastern highlands.

## Discussion

This study analysed projected changes in climate extremes across regions of Tanzania in the present (2011–2040), middle (2041–2070) and late (2071–2100) centuries under the RCP4.5 scenario. This study aimed to tailor a regional climate analysis that can be applied to strengthen resilience and reduce societal vulnerability to anticipated climate extremes by developing well-informed and actionable adaptation strategies and practices.

The data used in the analysis of climate extremes were drawn from the outputs of high-resolution regional climate models that are included in the Coordinated Regional Downscaling Experiment Program (CORDEX-Africa). However, the Regional Medels included in the CORDEX system fail to capture the climate of Tanzania. For instance, over areas that receive bimodal patterns of rainfall in Tanzania, CORDEX RCMs overestimate rainfall in short rainfall seasons (October-November-December) and underestimate the amount of rainfall in the main rainfall season (March-April-December) (see Fig. 2). The failure of CORDEX RCMs to represent the climate of Tanzania reasonably motivated the author to use a statistical downscaling technique, the delta downscaling method, to adjust the model simulations, which is in agreement with^26^, who argued that the results from RCMs often fail to represent the local or regional climate and that statistical empirical schemes must be imployed to refine the data to obtain a realistic regional or local representation of the climate.

Based on the presented and analysed results, projections show that Tanzania is experiencing and will continue to experience climate extremes related to both temperature and rainfall in all projection periods under the RCP4.5 scenario. The projections of the number of warm days (TX90p) are spatially coherently increasing throughout the country but are more rapidly warming in the eastern and northern regions by approximately 100 to 300 days for all projected times under RCP4.5. The number of cold days (TX10p) is projected to decline across regions of Tanzania and more rapidly warm over colder areas such as the southwestern and northeastern highgrounds of Tanzania. These findings imply that, in Tanzania, climate extremes could have contributed to the existing outbreak of new diseases and pests and changes in biodiversity and ecology that have been reported from areas that used to have colder climates but are currently experiencing increasing warming. There is, for instance, an increase in the incidence of multiplication of insects on the southwestern high ground, which could be attributed by increased temperature, which increases the rate of hatching of eggs from insects. The plants and vertebrates could have been impacted by increasing temperature, as temperature shortens the length of growing seasons of plants and decreases crop yields in some crops. In the future, climate temperatures are projected to continue increasing, increasing the impacts to social livelihood of people across many regions of Tanzania. Therefore, the presented results call for immediate adaptation to increased temperatures across regions of Tanzania.

Projections of rainfall revealed that under the RCP4.5 scenario, few regions across Tanzania feature consecutive dry days (CDDs). However, a large part of Tanzania is projected to experience a decrease in consecutive dry days. This is a good way for farmers to prepare for adaptation measures by using opportunities created by climate change. On the other hand, consecutive wet days are likely to increase in a few regions of Tanzania and will remain unchanged and decrease in many regions across Tanzania.

The projection of climate extremes related to extreme rainfall reveals that regions in Tanzania are likely to continue experiencing heavy, very heavy and extremely heavy rainfall. The entire coastal region, southern and northwestern regions and southwestern and northeastern highlands are likely to experience extreme rainfall that can continue to contribute to flood events that have caused significant socioeconomic losses and death. These results call for relevant authorities to strengthen adaptation measures against extreme rainfall, particularly throughout the entire coastal region and southern and northeastern highlands.

## Conclusion and recommendation

In this study, we presented an analysis of climate extremes in Tanzania using climate-simulated data from high-resolution regional climate models included in the Coordinated Downscaling Experiment Program (CORDEX-Africa). The innovation of this study is to further downscale the output from RCMs using the delta method and regrid the entire dataset from 0.44° by 0.44° spatial resolution to 0.05° by 0.05° spatial resolution. Projections of climate extremes in the present, mid- and end-century under RCP4.5 were analysed. The findings reveal that regions across Tanzania will continue to warm as a result of climate change, and colder places are projected to warm faster than warm areas. The nights will continue to warm, which could contribute to increased sleep discomfort for people living in urban areas where warm nights are predominantly anticipated. Heavy to exceptional heavy rainfall will continue to occur in the future climate, predominating throughout the entire coastal region and southwestern and northeastern highlands. The results presented here call to relevant authorities for the immediate strengthening of climate extreme adaptation related to both temperature and rainfall.

## Data Availability

The datasets used and/or analysed during the current study available from the corresponding author on reasonable request.
